# Measuring real-time cognitive engagement in remote audiences

**DOI:** 10.1038/s41598-023-37209-7

**Published:** 2023-06-29

**Authors:** Ana Levordashka, Danaë Stanton Fraser, Iain D. Gilchrist

**Affiliations:** 1 Department of Psychology, University of Bath, Bath, BA2 7AY UK; 2 School of Psychological Science, University of Bristol, Bristol, BS8 1TU UK

**Keywords:** Psychology, Human behaviour

## Abstract

Responses to arts and entertainment media offer a valuable window into human behaviour. Many individuals worldwide spend the vast majority of their leisure time engaging with video content at home. However, there are few ways to study engagement and attention in this natural home viewing context. We used motion-tracking of the head via a web-camera to measure real-time cognitive engagement in 132 individuals while they watched 30 min of streamed theatre content at home. Head movement was negatively associated with engagement across a constellation of measures. Individuals who moved less reported feeling more engaged and immersed, evaluated the performance as more engaging, and were more likely to express interest in watching further. Our results demonstrate the value of in-home remote motion tracking as a low-cost, scalable metric of cognitive engagement, which can be used to collect audience behaviour data in a natural setting.

## Introduction

Art and entertainment media elicit powerful shared experiences^1–6^, play a crucial role in psychological development^7^ and quality of life^8^. Moreover, they are an activity individuals engage with readily and frequently^9^. In the United Kingdom, the average amount of time a person spends watching video content was recently estimated to be 5 h 16 min per day^10^. Understanding the preference and viewing behaviour of audiences is key to developing and evaluating creative content^11^ and offers a window into neural and psychological processes^12–15^. The complexity of audience experience, as well as the apparent ease and willingness with which individuals engage with creative content, are in stark contrast with the commonly available research methods and there is a pressing need to develop non-invasive, scalable, cost-effective measures of audience response^16,17^. As most media content is consumed at home, there is a particular need for measures, which can be administered remotely.

Audience research typically relies on viewership statistics, retrospective surveys, interviews and laboratory studies. Viewership statistics and ticket sales can be obtained automatically and unobtrusively and at a large scale, including nationally-representative samples. They have high ecological validity but are crude and offer little insight into underlying processes involved in engagement. With the growing tendency to multi-task with devices, having content playing at home does not mean actual engagement^18^. Retrospective surveys and interviews are a commonly-used method for gaining deeper understanding. While certainly useful, retrospective self-reports have limitations, including sampling and recall biases, and may systematically exclude individuals who are not able to verbalise and reflect on their experiences^16^. Importantly, they reveal little about the temporal dynamics of the audience members response^13^. Laboratory studies can provide unique insight into underlying processes through in-depth analysis triangulating behavioural and neurophysiological measures^1–3,6,13–15,19^. Due to the extensive amount of resources and expertise required, laboratory research tends to be costly and conducted with smaller, convenience samples. With few notable exceptions^3,6,15,20^, this work is primarily constrained to laboratory settings. The present research seeks to extend these methodologies by using automated motion tracking to remotely measure real-time engagement in a large sample of home audiences. Measuring overt behaviours, such as movement, is a promising opportunity for a cost-effective, non-invasive, and scalable measure of engagement, provided that these behaviours can be reliably linked to subjective experiences.

The relationship between movement and engagement in screen-based media is a topic that has attracted increasing attention in recent years. Research in seated audiences suggests that prolonged stillness and blank facial expressions may be indicators of enjoyment and engagement, which is in line with earlier observational studies^21,22^ and supports what is known as the Stillness Hypothesis^20^. There are theoretical reasons to expect reduced movement during engagement in passive viewing. The narrowing of attention^13,23^ could lead individuals to neglect content-unrelated information, including physical discomfort, which would normally cause fidgeting. There is research linking selective attention to parasympathetic activity—a state of lowered heart rate and bodily relaxation^24^, which is consistent with evidence of lowered heart rate during media engagement^4,25,26^. Mind-wandering, a widely researched phenomenon that involves shifting attention away from a primary task toward internal information, such as memories, has been linked to movements in the face and body. Mind-wandering can be unrelated or related to the task or stimulus. While task-unrelated thought indicates low engagement, task-related thought can be seen as engagement in the form of “reflection” or “sense-making”. Regardless of its content, mind-wandering supposes reduced attention to primary content and can be expected to present as such on objective measures, including reaction times^13,19^ and movement^20^.

Based on this relevant literature, we distinguish between four different levels, or types, of engagement: (a) distracted or disengaged, that is, not paying attention to content; (b) attentive or simply engaged, that is, not distracted, but not deeply focused; (c) reflecting, or thinking about the content, similar to task-related mind-wandering; (d) immersed, or deeply engaged. Immersion is a form of deep engagement, defined as a “state of deep mental involvement, accompanied by reduced awareness of the physical world^27^”, Characterised by heightened perceptual awareness of the primary stimulus and reduced awareness of extraneous information, including the passing of time, immersion is comparable to the state of flow^28^. According to this definition, immersion is theoretically distinct from related concepts, such as presence^29^, system immersion as an objective property of technology^30,31^, and immersive theatre or experiences^32^. “Reflection” or “sense-making” can be thought of as task-related mind-wandering. Grounded in relevant theory, the typology we introduce provides a more nuanced understanding of how movement relates to engagement.

Beyond a direct relationship between movement and engagement, motion tracking can be used to examine complex associations and improve precision. The method is suitable for micro-analysis, an informative approach, which has led to major discoveries in the social sciences^33,34^. Computationally lightweight crowd tracking^35^ is well-suited to examining the dynamics of audience behaviour at scale. Advances in computer vision, such as the detection of microscopic movements^36^, make it increasingly possible to examine physiological signals like heart and respiration rate. These technologies, combined with modelling techniques, have the potential to disambiguate between attentional states and produce precise, reliable metrics.

To measure continuous audience engagement in a remote, unmoderated setting, we developed a bespoke web-based application, which allowed us to obtain real-time face-tracking and experience-sampling data, whilst streaming video content. The primary aim of this research was to test whether movement predicted audience engagement. A key hypothesis emerging from the literature is that the total amount of movement should be negatively associated with engagement. A secondary aim was to gain a more precise understanding of the relationship between movement and different forms of engagement and specifically whether immersion, the state of deep engagement and heightened perceptual awareness, can be distinguished from mere engagement (attentiveness) and reflection (task-related mind-wandering). Lastly, to test whether the presence of experience-sampling negatively impacts overall experience, we introduced an experimental manipulation whereby half of the participants were randomly assigned to watch the video without any mention of engagement.

We preregistered three hypotheses^37^. First, as an initial validation of the approach, we hypothesised that the two momentary measures, Head Movement and Momentary Engagement, would be temporally synchronised across participants, that is, there would be times in the performance where participants tended to respond and behave in a similar way (H1). Such correspondence should be reflected in positive inter-subject correlations. Inter-subject correlations (ISCs) are a method of measuring the consistency of responses to complex, naturalistic stimuli across individuals^38^, typically used as a complementary analysis. Correlations are computed for each pair of subjects and the distribution of r values is statistically compared to a null distribution with a mean of zero. Second, the main study hypothesis was that immersion is associated with reduced head movement (H2). We additionally hypothesised that the inclusion of an experience sampling measure would not undermine engagement, that is, there would be no significant difference in retrospective engagement between the experience sampling and viewing-only condition (H3).

**Figure 1:**
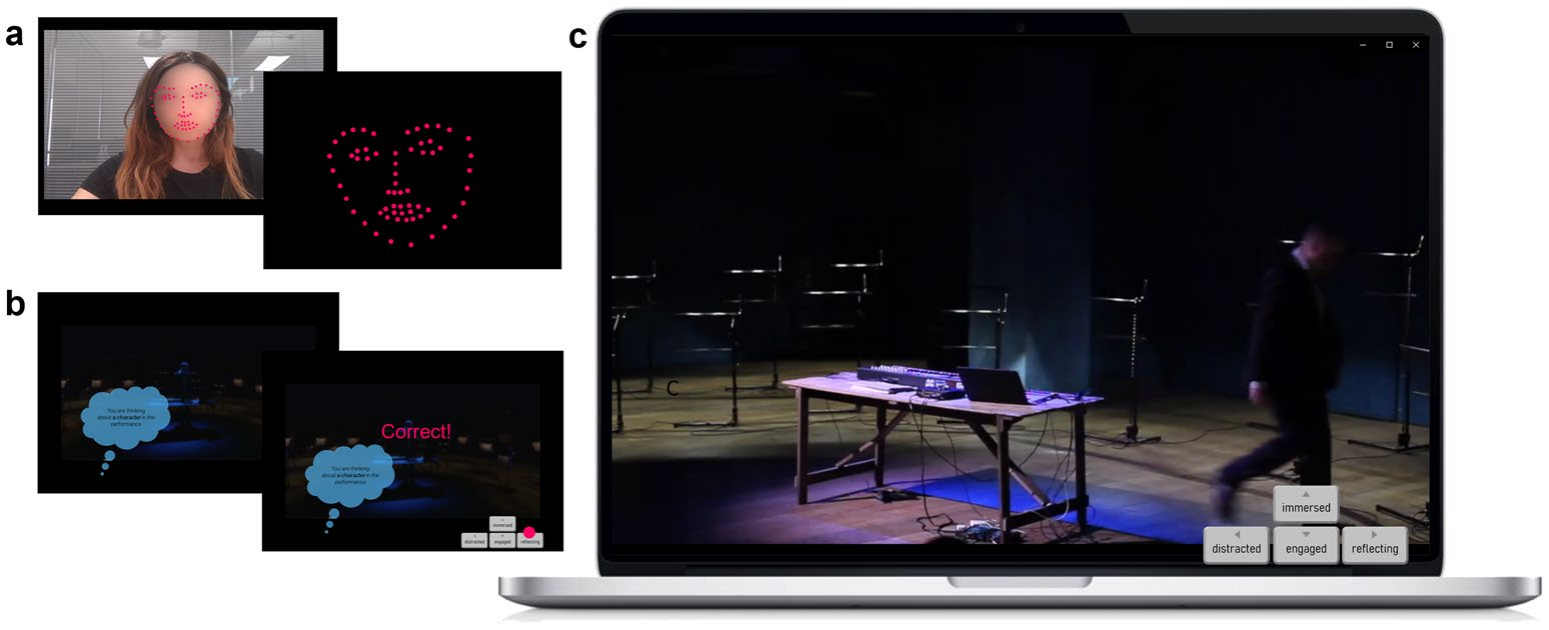
Illustration of key moments in study flow. (**a**) Face-Tracking calibration. Participants were presented with the preview of the face tracking algorithm with facial coordinates overlayed over their web-camera input in real time, followed by a screen showing only their facial coordinates; (**b**) Training trials for reporting the momentary engagement in Experience Sampling Condition; (**c**) Media Player as seen by participants, featuring a still from the stimulus play The Bullet and The Bass Trombone and response scale visible. Participants in the Control Condition were not presented with training trials (**b**) and response scale (lower right).

The research took place online via a standard web-browser, in a bespoke interface (Fig. 1). Participants (N = 132) watched a 30-min segment of the theatre play “The Bullet and The Bass Trombone” by sleepdogs^39^, a contemporary piece, combining minimal visuals and a rich soundtrack. The piece was selected to elicit different levels of engagement across time and individuals due to its intricate weaving of sound and narrative. Further details on the piece are provided in the Methods. The sample was gender-balanced (50% female; 2 non-conforming) and ethnically diverse, with 67% self-identifying as White. Mean age was 30 (SD = 11).

With participants’ permission, we obtained a web-camera recording of their head and shoulders alongside real-time face tracking. As hand movements are typically not present in the view when a web camera is used in a natural position, we focused on head movements. There were two experimental conditions to which participants were randomly assigned: One group of participants just watched the performance (Control condition, n = 58) and the other group reported engagement during viewing (Experience-Sampling Condition, n = 74). The Experience Sampling Condition included a measure of momentary engagement, based on established measures of probe-caught mind-wandering^40^. Participants used the keyboard to report their level of engagement during viewing in response to 23 sound probes, approximately 1-min apart, using the categories: *distracted*, *engaged*, *reflecting*, *immersed*. At the end of the segment, all participants completed a questionnaire, measuring their retrospective engagement. Additional details can be found in the Methods section. The research, design and hypotheses were preregistered. The study materials, anonymised data, analysis scripts, and preregistration documentation can be accessed on the Open Science Framework^37^.

## Results

### Variable descriptives

#### Head movement

Head Movement was operationalized as the second-by-second change in middle of head position based on the face tracking marker associated with the tip of the nose. We used the measured interocular distance from the face tracker markers to rescale the movement to centimetres. Head Movement, measured in centimetres was right-skewed (Skewness = 5.74, Kurtosis = 47.7) with Mean = 0.67 (*SD* = 0.67%, range 0–19.97 cm). The average Head Movement registered during a 1-s time period was 0.67 (*SD* = 1.43 cm).

### Momentary engagement

Momentary Engagement is the response received to each of 23 engagement probes. This measure achieved good variability. The most common response type was engaged (36%), followed by immersed (24%), reflecting (23%), and distracted (17%). The majority of participants (81%) used at least three of the four response types at least twice; 24% used all four responses at least twice. Only two participants gave the same response to the majority of probes (80% or higher).

#### Retrospective engagement

The retrospective evaluation questionnaire included measures of engagement (Narrative Engagement Questionnaire^41^) and enjoyment (items from the Immersive Experience Toolkit^42^). Items in each scale were highly correlated and the scales are therefore treated as single scores. Engagement in the sample was moderately high (*M* = 3.3, *SD* = 0.8 on a scale ranging from 1 to 5). The average Enjoyment was 62 (*SD* = 26, range 0–100). The two scores were highly correlated (r = 0.72, p < 0.001).

In a bespoke measure of behavioural intention, participants were asked whether they would have liked to continue watching, with the clarification that they would not be asked to do so regardless of their answer. The majority of participants (*n* = 49; 66%) responded “Yes”. Participants who wanted to continue watching scored higher on Narrative Engagement (*M* = 3.6, *SD* = 0.7) compared to those who did not (*M* = 2.6, *SD* = 0.6; t-test, t(91.53) = − 9.42, p < 0.001).

### Measure validity

#### Momentary measures are temporally correlated across participants

For Head Movement, we only considered inter-subject correlations (ISCs) in the Control Condition, since the Experience-Sampling Condition included probes played at fixed times, potentially introducing temporally coordinated movement associated with the probes and responding to them and so inflated ISCs. As preregistered and in accordance with the literature on ISCs, we calculated a moment-by-moment correlation for each pair of participants and tested if this distribution of correlation coefficients was greater than zero, where zero would mean no temporal coherence between responses.

The analysis included 58 participants, or 1652 unique pairs. The pre-registered one-sample t-test revealed that the observed r values (mean *r* = 0.005) is significantly larger than a distribution with a mean of zero, t(1651) = 4.06, p < 0.001, n = 1652. To check whether the presence of participants who made little to no physical head movement (i.e. were very still) affected the ISC, we repeated the analysis after excluding participants in the lowest quartile of mean and standard deviation in Head Movement, analysing data from 44 participants, or 253 unique pairs. The average correlation was similar in magnitude (*r* = 0.005) and despite the over six-fold reduction in sample size, the distribution’s difference from zero remained significant, t(944) = 2.6, p = 0.0095, n = 945.

For the categorical Momentary Engagement, we calculated non-parametric correlations for each response category using dummy variables. For each analysis, the response category was coded as 1 and all other categories as 0. Inter-subject correlations were significant for all categories, with the exception of ‘engaged’, which was approaching significance: distracted, t(1860) = 3.57, p < 0.001, n = 1861; reflecting, t(2374) = 2.79, p = 0.005, n = 2375; engaged, t(2591) = 1.51, p = 0.13, n = 2592); immersed, t(1824) = 3.23, p = 0.001, n = 1825. These results suggest that there were times in the performance at which participants self-reported engagement were consistent beyond chance level.

#### Effect of experience sampling on engagement

To examine whether the inclusion of an experience sampling measure influenced participants’ self-reported engagement, we tested the difference in Narrative Engagement between conditions using a Welch Two Sample t-test. The test was statistically non-significant (mean in group Control Condition = 3.39, mean in group Experience-Sampling Condition = 3.26, t(127.05) = 0.98, p = 0.327). A similar pattern emerged for Behavioural Intention–the percentage of people who reported willingness to continue watching was identical across conditions (66%).

### Stillness hypothesis

**Figure 2:**
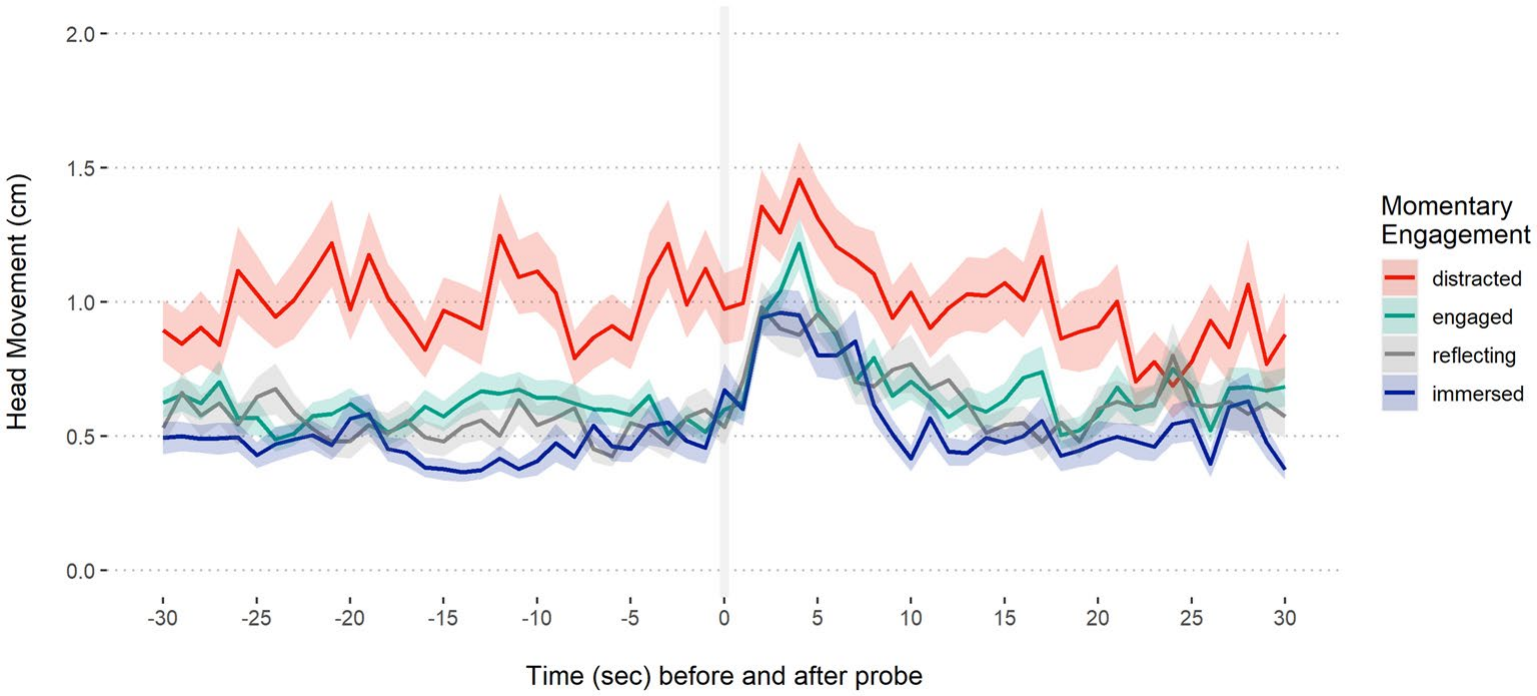
Head movement and momentary engagement. Head movement in the 30 s before and after a given probe as a function of reported momentary engagement type. Ribbons represent standard errors.

**Figure 3:**
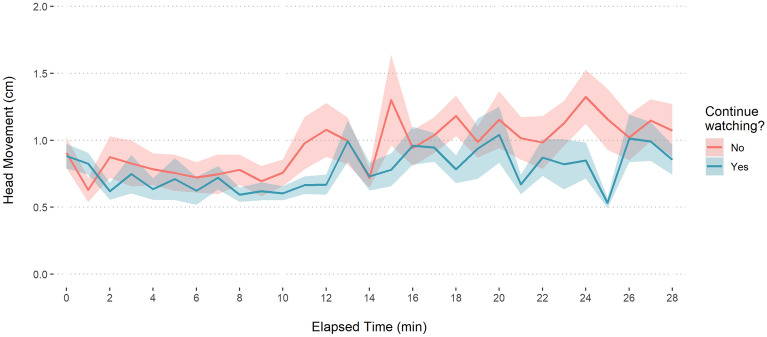
Head movement and retrospective engagement. Head movement over the course of the performance, aggregated by minute, as a function of willingness to continue watching. Ribbons represent standard errors.

We specified and pre-registered two directional hypotheses on the relationship between movement and immersion: a response of ‘immersed’ on the Momentary Engagement measure would be preceded by less Head Movement, that is, greater stillness; Retrospective Engagement: the average score on the Narrative Engagement Questionnaire, would be negatively associated with Head Movement. Before reporting the pre-registered analysis, where Head Movement was aggregated, we examined the effects in the full time-series data. Figures 2 and 3 display movement (Head Movement) over time, as a function of momentary and retrospective engagement, respectively.

As can be seen in Fig. 2, individuals reporting being distracted moved significantly more before and after receiving a response prompt. All forms of engagement produced a spike in movement immediately following the prompt, which then returned to pre-prompt levels. The spike in movement may have been caused directly by the act of responding, which was carried out using a keyboard press. Two noteworthy patterns with regards to immersion are the relatively prolonged period of stillness (10 s starting 20 s prior to the probe) and the relatively steep return to stillness following the probe. Figure 3 shows that participants who moved less over the course of the performance were also the ones who reported willingness to continue watching.

#### Stillness and momentary engagement

To test whether ‘immersed’ responses were preceded by greater stillness, we conducted paired-sample t-tests. Momentary Engagement response type (immersed, engaged, reflecting, distracted) was provided by each Participant on each Probe, resulting in nested data. We aggregated responses by Probe, calculating the average Head Movement associated with each Probe. Probes with less than 3 data points were excluded as their mean values were likely to be influenced by outliers (*n* = 2 Probes; 0.1%). Due to significant deviation from normality (Shapiro–Wilk normality test, W = 0.95, *p* = 0.044), we conducted non-parametric Wilcoxon signed rank test on paired samples.

**Figure 4:**
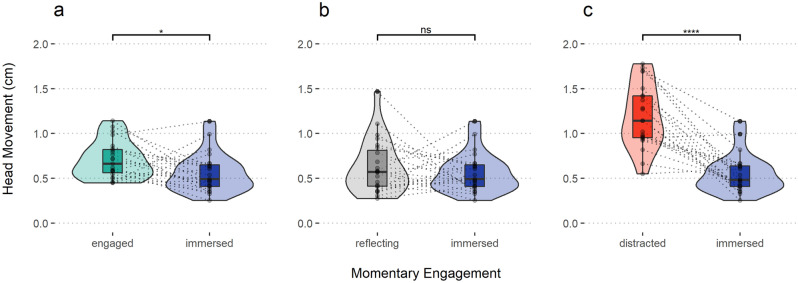
Paired T-tests: head movement and engagement type. Difference in mean head movement between response types. Each point represents one probe (time point in the performance when experience was sampled). Boxplots denote median and quartiles, violin plots provide density estimates.

We conducted pairwise tests for each pair of categories, correcting for multiple family-wise comparisons (Bonferroni correction). As can be seen in Fig. 4a–c, responses ‘immersed’ were preceded by less Head Movement, as compared to responses ‘engaged’ (Wilcoxon test, W = 211, p = 0.026, n = 46) and ‘distracted’ (Wilcoxon test, W = 250, p < 0.001, n = 44). The difference between responses ‘immersed’ and ‘reflecting’ was non-significant (Wilcoxon test, W = 157, p = 0.58, n = 46).

#### Stillness and retrospective engagement

The Spearman’s rank correlation rho between Head Movement and Retrospective Engagement was in the predicted direction–higher score on Narrative Engagement was associated with lower Head Movement- and statistically significant (rho = − 0.27, S = 485,789, p = 0.002).

For the binary Behavioural Intention, participants who reported willingness to continue watching, moved significantly less overall (*M* = 0.68, *SD* = 0.41) than those who did not (*M* = 0.96, *SD* = 0.84). To better reflect the temporal variability of movement, we conducted an analysis comparing minute-by-minute engagement in each group, using a paired-samples t-test. The effect was significant: Wilcoxon test, W = 26, p < 0.001, n = 58.

**Figure 5:**
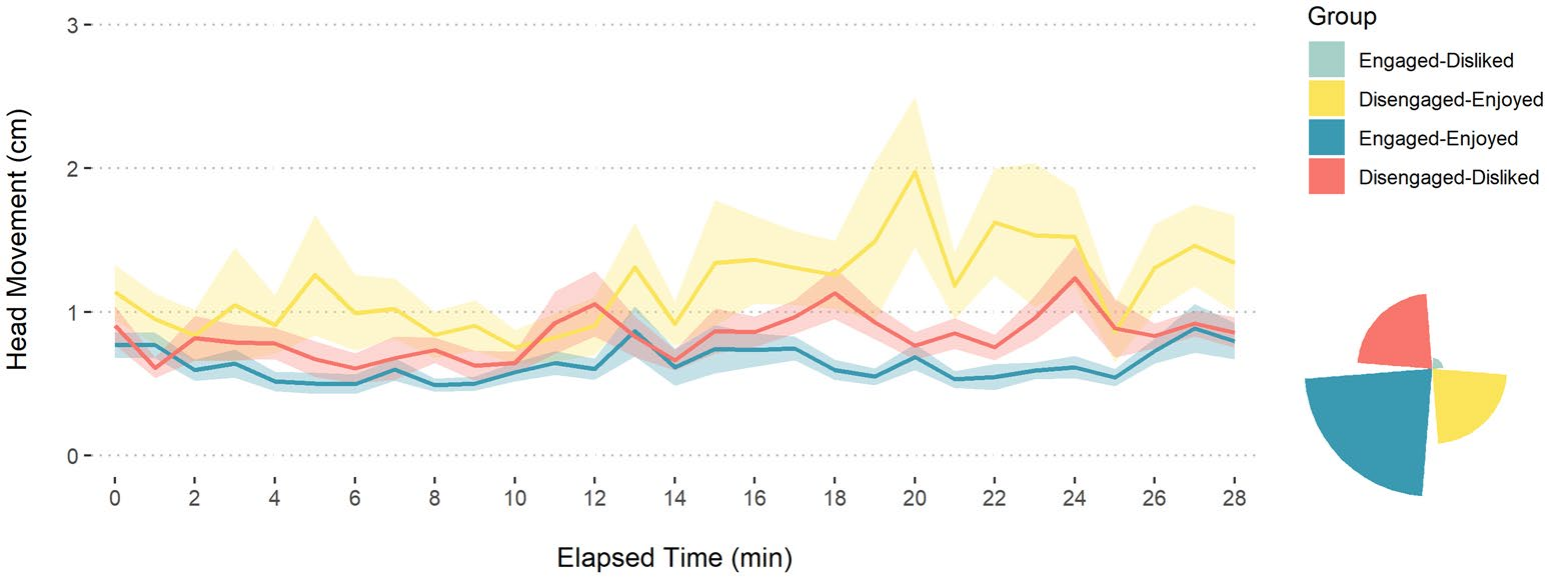
Movement across participant groups based on Retrospective Engagement and Enjoyment. Circumplex chart (bottom right) represents sample size of each group. Line graph (top left): Lines represent minute-by-minute mean Head Movement; Ribbons are Standard Errors. Group ‘Engaged-Disliked’ not included in the line graph due to limited number of observations (n = 5).

There was no significant correlation between Head Movement and Enjoyment (rho = − 0.06, p = 0.487). This was surprising, considering the high positive correlation between the two outcome variables, self-reported Engagement and Enjoyment (*r* = 0.72). To better understand this discrepancy, we looked at participants deviating from the correlation. Four groups were considered: Participants who scored high, that is, above the median, on both Engagement and Enjoyment (Engaged-Enjoyed, *n* = 58), low on both (Disengaged-Disliked, *n* = 34), and the two discrepant scores (Disengaged-Enjoyed, *n* = 34; Engaged-Disliked, *n* = 5; the final group was not included in the analysis due to low size). Group size and movement profile can be seen in Fig. 5.

Comparing movement across these groups (Fig. 5), reveals the expected relationship between movement and engagement, whereby participants who reported being engaged and enjoying the performance moved less than those reporting low engagement and enjoyment. According to pair-wise comparisons of minute-by-minute movement in each group, these differences were significant (Wilcoxon test, W = 17, p < 0.001, n = 58). Participants who reported low engagement and high enjoyment, exhibited an even higher degree of movement (Wilcoxon test, W = 422, p < 0.001, n = 58).

## Discussion

The aim of this research was to measure real-time cognitive engagement in video content in a remote unmoderated setting. Existing measures of real-time engagement typically require laboratory tasks and equipment. The new method we developed and report here allowed us to measure real-time engagement from over a hundred individuals across the world. This was successfully accomplished through face tracking embedded in a web-based environment, requiring only a web camera and internet connection.

In a systematic investigation of 132 participants, we provide evidence in support of the hypothesis that movement is negatively associated with engagement in seated audiences. The association was robust and consistent across measures, including probe-caught momentary engagement, retrospective report of overall engagement, and intention to continue watching. Individuals who moved less reported feeling more engaged and immersed, evaluated the performance as more engaging, and were more likely to express interest in watching further.

The present research draws on the Stillness Hypothesis and the theoretical understanding that cognitive immersion leads to a narrowing of attention and served as a basis of the metric we proposed. Evidence for the association between stillness and engagement was found in research measuring the hand movements of live audiences^20^. That we observed an analogous relationship when considering head movements during private viewing suggests an internal underlying process, corroborating theories linking engagement to relaxation^4,24^ and the narrowing of attention^12,13^. In accordance with this theory, we would expect that measuring head and hand movements are complementary, with each being a reliable indicator of fidgeting behaviour and their combination capturing more instances. Similarly, being in a live audience can be expected to heighten the effect due to behavioural contagion. Even if we cannot fully rule out the possibility that participants’ knowledge of being recorded impacted their behaviour and consider the context strictly private, it is still plausible that a public setting would amplify the effect. These are intriguing questions for future research. To gain a nuanced understanding of how movement relates to cognitive engagement we measured multiple constructs. The results suggest that head movements may primarily reflect fidgeting corresponding to disengagement, rather than more subtle processes such as thinking and deep perceptual immersion. Participants who reported being distracted at a point in time moved nearly twice as much as those who were attentive. A somewhat unexpected result was the similarity between immersion ("fully immersed in the performance losing track of time") and reflection (“thinking about the performance, story, characters, sounds, personal associations”). That we did not capture a strong difference between these two types of response is an indication that head movements, here at least, correspond to more pronounced forms of distraction.

Although the dissociation was less pronounced, the movement profiles associated with each engagement state were not identical. The difference between immersion and reflection was in the expected direction and approaching significance. The difference to mere engagement ("primarily paying attention to the performance, not thinking about or doing anything else") was small but significant. These dissociations suggest that a more fine-grained operationalisation of movement involving facial and postural features, may be able to discriminate immersion from other forms of engagement.

An initially surprising result was that despite a strong positive correlation between self-reported engagement and enjoyment of the presented content, and a positive association between movement and engagement, there was no association between movement and enjoyment. The discrepancy was due to a group of individuals who reported low engagement and high enjoyment and displayed a substantial degree of movement. Understanding the exact characteristics and motivations of this audience group warrants further investigation but was beyond the scope of the present research. Crucial here is that movement did correspond to individuals' self-reported willingness to continue watching, making movement a valuable, resource-effective tool for insight into audience behaviour.

The premise remains that for accurate assessment, especially if individual-level precision is desirable, overt responses may need to be disambiguated. We demonstrate that this can be accomplished through experience sampling. Our experimental manipulation demonstrated that inclusion of an experience sampling measure did not appear to interfere with overall engagement and enjoyment. This finding speaks to the validity of our approach—if the momentary measures had interfered with audiences’ experience of the context, our conclusions would be less valid. It also shows that asking for audience feedback during a performance is not overly disturbing, at least when carried out in a subtle, intuitive way.

The results also confirm that participants were able to recognise and report subjective engagement states, including immersion, engagement, reflection and distraction. Participants were able to understand the distinction in our definition and examples and used all categories when reporting their experienced states. Currently, validated self-report categories revolve around liking^43^ or valence—arousal^44^. Our research contributes a set of attention-oriented response categories: “distracted, engaged, reflecting, and immersed”.

The present research was carried out using a single content piece—a contemporary theatre performance which featured monologue, storytelling, and immersive soundscapes, which we chose deliberately in order to produce very wide levels of engagement across time and individuals (the success of which is evident in the data) and to expose audience members in our study to unfamiliar content. Based on the theoretical underpinnings of the Stillness Hypothesis, we expect our findings to generalise to other content with predominantly visual or narrative components. For music, especially dance music, different approaches may be required, for example interpersonal synchrony. It is possible that certain aspects of the piece contributed to the results. However, the study's focus was on method development, rather than investigating the specific effects of this content on audience engagement. Future work can build on our work to validate and refine movement-based measures across different types of content and audiences, while retaining the ecological validity that comes from watching an extended piece of content. An important direction for future research would be to produce a more fine-grained operationalisation of movement. The current operationalisation is based on head movement in two dimensions. A more comprehensive quantification could include estimation of three-dimensional motion, tracking of shoulders, hands, face, and temporal dynamics, such as sudden vs rhythmic movements. Our results suggest that it may be possible to devise movement-based measure of deep immersion, as distinct from attentiveness. Doing so may require additional measures, including physiology and inter-personal synchrony, which are better suited than self-report for detecting immersion, which is characterised by a lack of meta-awareness.

In conclusion, the present research presents the use of head movement as a cost-effective, scalable metric of engagement. Computationally, head tracking is simpler and more reliable than full posture estimation and therefore applicable in large, dense audiences, such as festival crowds, where only heads are visible; or in private homes, where collecting more fine-grained data can be ethically problematic. The metric can be fully anonymous and therefore suitable for crowd-sourced data collection. It can, for example, be embedded in online media players or TV set meters, to improve the validity of viewership statistics. Apart from applications in research, the metric can be used in creative production, for example, in interactive real-time visualisations audiences in virtual productions.

Beyond audience research, our results contribute to an important body of work demonstrating that attentional states can be observed. While early studies relied on painstaking frame-by-frame manual coding, emerging motion-tracking offers the possibility of instant large-scale quantification^35^ or ultra-fine-grained resolution^36^. Our research demonstrates that this technology can already be incorporated into research as a valuable tool for tracking mental states. Our research media player with face-tracking functionality is publicly available (RMPL: https://gitlab.pavlovia.org/alvd/trace).

## Methods

### Participants

Participants were recruited from the online research panel Prolific (https://www.prolific.co/). All participants provided informed consent and were paid £8 per hour. The research was performed in accordance with the Declaration of Helsinki and approved by The University of Bath Department of Psychology Research Ethics Committee (20-202). Participants with incomplete data, including fewer than 600 motion tracking points (n = 5) and 10 experience sampling responses (n = 5) were excluded from the analyses involving the respective variables.

### Performance

We showed the first 30-min of the play The Bullet & The Bass Trombone by sleepdogs, recorded in the Bristol Old Vic Theatre. In the play, a single actor plays an unnamed composer, telling the story of what happened to his orchestra during a military coup in a foreign country. We chose this piece because of its intricate weaving of sound and narrative: At times the performer falls silent, listening to a bird song, speaks over a cacophony of voices, or plays a recording of other actors telling their characters’ stories in the first person. These elements were likely to elicit a broad range of responses and this style of content will be unfamiliar to most participants, as evident in the distributions achieved on response variables.

### Media player

A bespoke web-based media player was developed to stream content in a naturalistic setting whilst obtaining web camera recordings alongside real-time face-tracking and experience-sampling data. The Research Media Player was developed in Python and JavaScript and ran on the research platform Pavlovia (https://pavlovia.org/). Face tracking was obtained using face-api, a JavaScript API for face detection and face recognition in the browser implemented on top of the tensorflow.js core API. The algorithm returns 68 facial landmarks and 7 facial expressions.

### Head movement

Frame-by-frame data were down-sampled to 1 frame per second, taking the first or next available frame for every second. For each frame, we calculated the horizontal and vertical left and right eye position based on the mean of the 6 identified markers on each eye (markers 37–42 for the left eye and 43–48 for the right eye). This resulted in a single horizontal and vertical estimate for the centre of each eye. For each sample we then calculated an Inter Ocular Distance (IOD, in pixels) using the estimated centre for each eye. Head Movement is the change in position between each frame of the centre of the nose.

As the samples are 1 s apart and the average IOD in adults is 6.3 cm we can convert the Head Movement into cm/s units using the sample-by-sample IOD in pixels. We define Head Movement on the first sample as 0. Samples where the Head Movement is greater than 20 cm/s were excluded as outliers (n = 0.2%). To aggregate movement over a period of time (a minute or a probe interval), movement was averaged across the number of seconds in the period.

### Experience sampling

While watching the performance, participants used the keyboard to report levels of engagement in response to audio probes (ca. 1-min apart). The measure was implemented in Pavlovia. Upon hearing the tone they used the keyboard arrow keys to indicate levels of engagement using one of the following categories:

- Immersed: fully immersed in the performance losing track of time and your surroundings
- Reflecting (task-related mind-wandering): thinking about the performance—story, characters, sounds, personal associations
- Engaged: primarily paying attention to the performance, not thinking or doing anything else
- Distracted (task-unrelated mind-wandering): disengaged, thinking or doing something unrelated to the performance

Participants learned the category definitions, alongside examples, and completed practice trials at the start of the study. The category labels were displayed in the bottom right corner of the screen throughout the performance, as shown in Fig. 1. The measure is based on probe-caught mind-wandering^40^. Participants’ ability to comprehend the instructions and response categories were pretested.

### Retrospective engagement

At the end of the segment, participants filled out an online questionnaire. Measures included the Narrative Engagement Questionnaire^41^ and the Global Experience and Cultural Value subscales from the Immersive Experience Evaluation Toolkit^42^, and a bespoke single-item measure of Behaviour Intention (immediately after the performance segment finished, participants were asked a Yes/No question as to whether they would have liked to continue watching; with the clarification that due to time constraints, they would not continue watching, regardless of their response).

There were several questions addressing participants’ subjective experiences of immersion, including qualitative phenomenological accounts of each category and a closed question on which elements of the performance participants felt immersed in (sound, images, story, imagined visuals). The qualitative measures are not included in the present paper. At the end of the questionnaires, participants reported basic demographics and individual differences.

### Experimental manipulation

The study included an experimental manipulation. Half of the participants were randomly assigned to a version of the study which included an experience-sampling measure. They were asked to report their engagement in response to sound probes presented during the performance, received definitions of different engagement types and completed practice trials. The remaining participants watched the performance with no interruptions and mention of engagement.

## Data Availability

Materials, data, and analysis scripts are available on the Open Science Framework at: https://osf.io/vxbj7/?view_only=8fbfca96a01e4d79ad50bbe448c03c65.
